# Association of a Network of Immunologic Response and Clinical Features With the Functional Recovery From Crotalinae Snakebite Envenoming

**DOI:** 10.3389/fimmu.2021.628113

**Published:** 2021-03-15

**Authors:** Charles J. Gerardo, Elizabeth Silvius, Seth Schobel, John C. Eppensteiner, Lauren M. McGowan, Eric A. Elster, Allan D. Kirk, Alexander T. Limkakeng

**Affiliations:** ^1^ Department of Surgery, Duke University, Durham, NC, United States; ^2^ DecisionQ, Arlington, VA, United States; ^3^ Department of Surgery, Uniformed Services University of the Health Sciences, Bethesda, MD, United States

**Keywords:** snake bite, antivenin, cytokine, prognostic model, predictive modeling, chemokine, Patient Specific Functional Scale

## Abstract

**Background:**

The immunologic pathways activated during snakebite envenoming (SBE) are poorly described, and their association with recovery is unclear. The immunologic response in SBE could inform a prognostic model to predict recovery. The purpose of this study was to develop pre- and post-antivenom prognostic models comprised of clinical features and immunologic cytokine data that are associated with recovery from SBE.

**Materials and Methods:**

We performed a prospective cohort study in an academic medical center emergency department. We enrolled consecutive patients with Crotalinae SBE and obtained serum samples based on previously described criteria for the Surgical Critical Care Initiative (SC2i)(ClinicalTrials.gov Identifier: NCT02182180). We assessed a standard set of clinical variables and measured 35 unique cytokines using Luminex Cytokine 35-Plex Human Panel pre- and post-antivenom administration. The Patient-Specific Functional Scale (PSFS), a well-validated patient-reported outcome of functional recovery, was assessed at 0, 7, 14, 21 and 28 days and the area under the patient curve (PSFS AUPC) determined. We performed Bayesian Belief Network (BBN) modeling to represent relationships with a diagram composed of nodes and arcs. Each node represents a cytokine or clinical feature and each arc represents a joint-probability distribution (JPD).

**Results:**

Twenty-eight SBE patients were enrolled. Preliminary results from 24 patients with clinical data, 9 patients with pre-antivenom and 11 patients with post-antivenom cytokine data are presented. The group was mostly female (82%) with a mean age of 38.1 (SD ± 9.8) years. In the pre-antivenom model, the variables most closely associated with the PSFS AUPC are predominantly clinical features. In the post-antivenom model, cytokines are more fully incorporated into the model. The variables most closely associated with the PSFS AUPC are age, antihistamines, white blood cell count (WBC), HGF, CCL5 and VEGF. The most influential variables are age, antihistamines and EGF. Both the pre- and post-antivenom models perform well with AUCs of 0.87 and 0.90 respectively.

**Discussion:**

Pre- and post-antivenom networks of cytokines and clinical features were associated with functional recovery measured by the PSFS AUPC over 28 days. With additional data, we can identify prognostic models using immunologic and clinical variables to predict recovery from SBE.

## Background

Snakebite envenoming (SBE) is a neglected tropical disease of global significance that primarily impacts low resource areas in low and middle income countries (LMICs), and snake-specific antivenoms are the cornerstone of therapy (1–8). Snake antivenoms have been used for over 100 years and have well demonstrated efficacy, but there remain significant safety, cost, and access to treatment considerations that limit their consistent use (5, 9). Currently available United States (US) antivenoms have a remarkable safety profile as compared to previous SBE biologics (10–16). Globally the safety profile of antivenoms is more mixed, and the risk-benefit profile of their use varies (17–19). This creates a potential barrier to their use in non-life threatening SBE where administration will not impact mortality, but can impact recovery and potential permanent disability (20).

Despite positive cost-effectiveness assessments of snake antivenoms, the cost to individual SBE victims can be beyond the financial means of many of those affected (21–23). This creates another barrier to use if the need for treatment is unclear due to the uncertainty of the severity and anticipated clinical course of the SBE patient (24, 25). There is also little economic incentive to pharmaceutical companies to develop and overcome the regulatory hurdles in order to distribute antivenom to where it is most needed (22, 26–28). This fact, combined with the lack of healthcare infrastructure in these low resource areas with a high prevalence of SBE, results in a lack of a consistently replaceable antivenom supply (28, 29). The concern for wasting antivenom when not absolutely needed, is accompanied by the risk of undertreatment for patients that would otherwise benefit (12, 30, 31). The described barriers to antivenom use are particularly important as treatment is time dependent with worse outcomes when care is delayed (32–38).

The clinical courses of SBE patients are highly variable, even within a single envenoming species, which further contributes to uncertainty regarding both the value of administering antivenom and how much is necessary for an individual patient (39). Unfortunately, there is no validated and reliable method of predicting severity or recovery in SBE (40). Historically clinicians have had to rely primarily on their own clinical experience. This judgement has been augmented by treatment guidelines and severity scales, but these have not been evaluated as to whether they improve clinical outcomes (7, 41–43). This has led to wide practice variation in when and how much antivenom is administered even within the US where safety and access to antivenom are much less of a concern (44). A prognostic tool that could predict the severity of SBE would be valuable in overcoming the stated barriers to antivenom use and allow for therapy tailored to the individual in order to prevent both under- and over-treatment. Hence, the need for novel prognostic strategies persists.

Snake venom is a complex mixture of enzymatic and non-enzymatic proteins and peptides. In the sub-family Crotalinae, the species *Crotalus horridu*s and *Agkistrodon contortrix* are known to have a large proportion of their venom composed of secretory phospholipase A2 (sPLA2), snake venom metalloproteases (SVMPs), and serine protease toxins. These toxins are of particular biologic importance and contribute to the venom-induced tissue injury, coagulopathy, and systemic symptoms seen in these SBEs (45, 46). However, the host inflammatory response is felt to also play a significant role in the pathophysiology and clinical syndrome of SBE (47–50). Consequently, evaluating inflammatory markers such as serum cytokines and chemokines give us a potential technique to evaluate immunologic response in SBE and hold promise as a component of a comprehensive prognostic strategy (48). Advances in enzyme-linked immune-assays using fluorescent bead technology now allow for simpler, reliable and rapid determination of cytokine levels (51). Consequently, the role of cytokines in other disease states is being investigated in LMICs using this technology (52–54).

Clinical features alone have not shown adequate prognostic performance in SBE, despite having some predictive value (37, 38, 40, 55, 56). Combining clinical features with immunologic markers has been used to prognosticate outcomes in other disease states (57–60). An improved understanding of the immunologic response combined with available clinical features in SBE could inform a prognostic model to predict severity and recovery in SBE. The purpose of this study was to develop pre- and post-antivenom prognostic models comprised of clinical features and immunologic cytokine and chemokine data administration that are associated with recovery from SBE.

## Materials and Methods

### Design

We performed a prospective cohort study at a single tertiary-care academic emergency department (ED) in the southeastern US in 2017 and 2019.

### Participants

We enrolled consecutive patients with acute snakebite envenoming (SBE) based on the Surgical Critical Care Initiative-enVenomation Investigation Pilot to Expedite Recovery (SC2i - VIPER) based on the SC2i - Tissue and Data Acquisition Protocol (TDAP) (*ClinicalTrials.gov Identifier: NCT02182180*). As the parent protocol has been previously described, we provide additional methods specific to the SC2i-VIPER sub-study that are necessary for reproducibility (57). We enrolled patients age 27 - 80 years presenting within the first 24 hours with verified acute SBE. No venom levels were obtained for enrollment, as all local venomous snakes are of the sub-family Crotalinae. Local species include *Agkistrodon contortrix* (copperhead) and *Crotalus horridus* (timber rattlesnake). Regionally transferred patients were eligible with the addition of the following potential Crotalinae species: *Crotalus ademanteus* (eastern diamondback rattlesnake), *Sistrurus miliaris* (pygmy rattlesnake) and *Agkistrodon piscivorus* (cottonmouth snake). Species identification was not required for enrollment, however the majority of investigator-identified species were copperhead snakes which predominate throughout the region. Exclusion criteria were pregnancy, prisoners, or inability to obtain consent during their stay for any reason. Informed consent was provided by a patient or proxy and the Duke University Institutional Review Board approved the study.

### Clinical Data

We assessed clinical variables as per SC2i protocol which includes static pre-existing variables such as sex, age, comorbidities and dynamic clinical variables such as laboratory results, medications, and vital signs. Clinical data were collected from patients and by review of the electronic health record using standard operating procedures.

### Biological Sample Collection and Processing

Samples from SBE patients were collected as soon as possible following their enrollment, ideally before antivenom administration when possible, as well as after initial antivenom administration. Whole blood was collected and serum was isolated at the site for the SC2i Consortium Biorepository following consortium-wide standard operating procedures and Guidelines for Good Clinical Laboratory Practice (GGCLP), and then stored until use for these analyses. The methods of sample collection and preparation have been reported, but are briefly encapsulated here for clarity (61).

### Sample Preparation

Frozen serum samples were thawed on wet ice for filtering. The samples could have a single freeze-thaw cycle. They were mixed and 400 uL were transferred to a 1.5 mL tube and centrifuged at 12,000g for 15 minutes at 4 degrees Celsius. A 350uL sample was transferred into a 0.65um filter tube (Millipore, Billerica, MA). They were centrifuged 12,000g for 30 minutes at 4 degrees Celsius. We then aliquoted 120uL of the filtered samples into clean tubes. We then flash froze samples in liquid nitrogen and stored in -80 degree Celsius freezer. The maximum time from freeze to thaw was less than one year.

After filtering, samples were thawed and a 1:50 dilution of samples were used. We measured levels of 35 unique cytokines using Luminex Cytokine 35-Plex Human Panel (Thermo Fisher Scientific, Waltham, MA) pre- and post-antivenom administration using standard cytokine preparation per kit instructions. A 1x wash preparation was created and used for all washes. Standard plates were prepared and 50ul of sample, standards, and incubation buffer were added to all wells and allowed to incubate for 2 hours. After washing, detection antibodies were added and incubated for one hour. After another wash, streptavidin-RPE was added and incubated for 30 minutes. These were washed again and beads were resuspended in wash buffer. For 35 plex analysis, we used Invitrogen Catalog# LHC6005M kits are from Thermo Fisher Scientific. The Comprehensive Human Cytokine Magnetic 35-Plex Panel provides reagents for the accurate, reproducible, and sensitive quantitation of human proteins including: EGF, Eotaxin, FGF-basic, G-CSF, GM-CSF, HGF, IFN-alpha, IFN-gamma, IL-1 beta, IL-1 alpha, IL-1RA, IL-2, IL-2R, IL-3, IL-4, IL-5, IL-6, IL-7, IL-8, IL-9, IL-10, IL-12, IL-13, IL-15, IL-17A, IL-17F, IL-22, CXCL10, CCL2, CXCL9, CCL3, CCL4, CCL5, TNF-alpha, VEGF.

### Outcomes

The Patient-Specific Functional Scale (PSFS), a well-validated patient-reported outcome of functional recovery, was assessed at 0, 7, 14, 21 and 28 days. Individual patient PSFS recovery curves over time were assessed to determine the PSFS area under the patient curve (AUPC) over the course of 28 days. The median PSFS AUPC was determined. Patients with PSFS AUPC great than or equal to the median were considered to have good recovery and those less than the median defined poor recovery.

### Data Analysis

For this preliminary analysis, we report baseline characteristics of all patients with available data. Continuous variables are summarized using mean, medians and interquartile range (IQR) as appropriate for the distribution of data.

To account for the small sample size with high dimensionality of the data set, we performed Bayesian Belief Network (BBN) modeling using a machine learning technique to build directed acyclic graphs that represent relationships with a diagram composed of nodes and arcs. Each node represents a cytokine, chemokine or clinical feature and each arc represents a joint-probability distribution (JPD). The JPDs were determined in a step-wise fashion over multiple iterations. We report associations of this cytokine/chemokine/clinical feature model with the patient’s recovery (PSFS AUPC). We built the BBNs employing FasterAnalytics Version 7.0. A BBN starts with an observation, then calculates the relationships of various variables that are likely to impact that observation based on known probabilities (beliefs). BBNs are very useful for illustrating the differences in the immune systems of different patient groups since they are represented by acyclic directed graphs. These graphs can be overlaid on each other so that we can easily see where there are differences in the cytokine, chemokine and clinical feature joint probability distributions. In this case, BBN’s can 1) identify which relationships exist between variables 2) allow us to measure the magnitude of relationships (one variable on another) but also the number of relationships for any given variable 3) handle a large number of variables without large datasets 4) compare which relationships exist and how many relationships exist between the two groups of recovery (good and poor). These models also deal easily with missing data, which was anticipated in this cytokine data set.

For the Luminex 35-plex kit, we built four Bayesian Belief Networks (BBN) for preliminary modeling using a Minimum Descriptive Lengths (MDL) of 0.01. The MDL measures the trade-off between goodness-of-fit and complexity of the model. Models with lower MDLs have more complexity and are more likely to be overfit. For each time point (pre- and post-antivenom), we built one model on the full data set and then models using leave-one-out. For each timepoint, we looked at the models and found which variables were first-degree associates of recovery. We used these variables in final modeling. If a variable was missing for more than 20% of records, we dropped the variable for the models with that kit.

We used leave-one-out cross validation. We used the scores from each test case to build a receiver operating characteristic (ROC) and measure the area under the curve (AUC). We consider any AUC of 0.6 or above to be a good differentiator between good and poor recovery. We also provided statistics about the connectivity of the models.

## Results

### Demographics

Twenty-eight snake envenoming patients were enrolled. Preliminary demographic data and PSFS AUPC is available for 24 patients with cytokine and chemokine data available from 9 pre-antivenom and 11 post-antivenom patients. The group was mostly female (82%) with a mean age of 38.1 (SD ± 9.8) years. **Table 1**.

**Table 1:** Demographics and clinical features of enrolled patients.

	N = 24	N = 11
Age -Min, Median, Max	25, 45, 78	27, 35, 56
Age - 1stQ, Mean, 3rdQ,	33.5, 46.2, 56	30.5, 38.1, 46.5
Sex (% Female)	17 (70.1%)	9 (81.8%)
White	22 (91.6)	10 (90.1%)
Asian	2 (8.3%)	1 (9.1%)
Comorbidity (at least one)	11(45.8%)	5 (54.5%)
Immunosuppressed	1 (4.1%)	0

### Recovery

**Figure 1:**
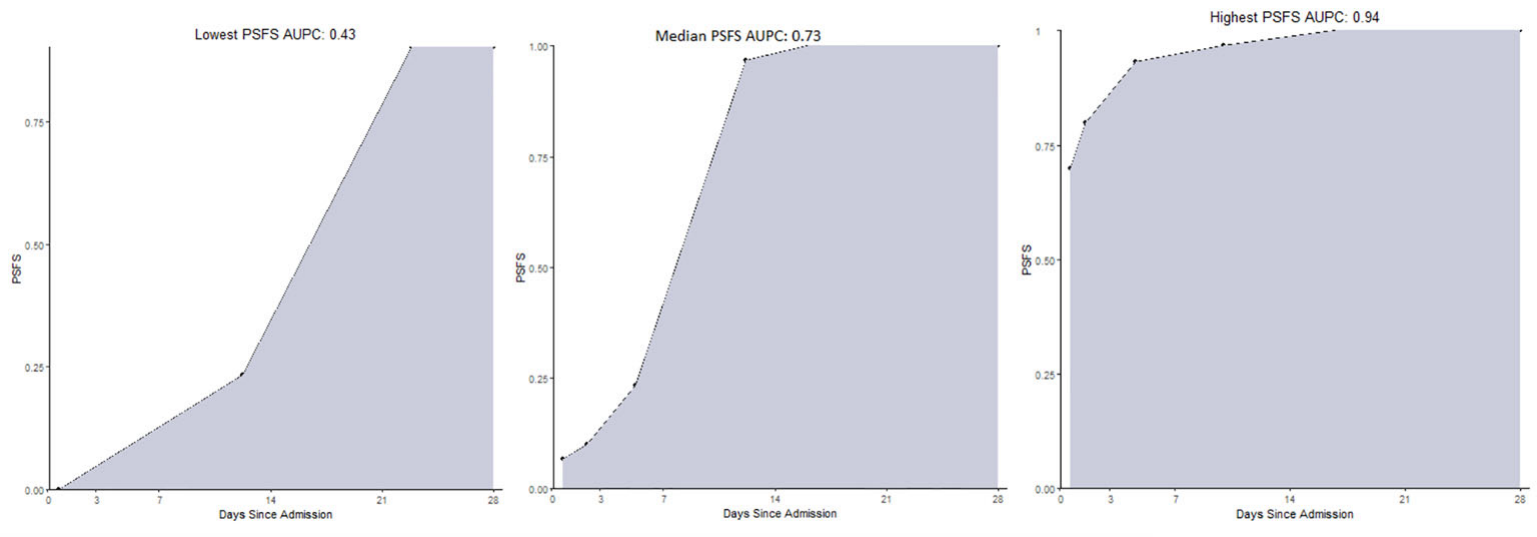
The range of the Patient-Specific Functional Scale Area Under the Patient Curves at 28 days for 24 patients with available preliminary data. (examples lowest, median, highest). PSFS AUPC, Patient-Specific Functional Scale Area Under the Patient Curve.

The patients’ functional recovery as measured by the PSFS AUPC, for the 24 patients with available data, had a median of 0.73 (range 0.43, 0.94) and is represented graphically in **Figure 1**.

### Pre- and Post-Antivenom Cytokines

**Figure 2:**
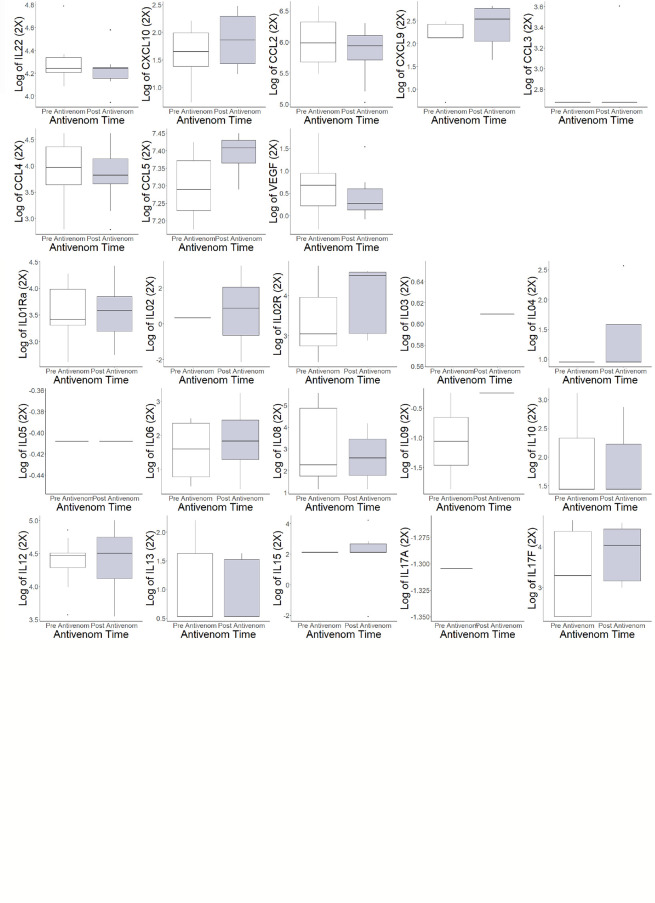
Individual cytokine and chemokine values pre- and post-antivenom treatment. EGF: endothelial growth factor, EOTAXIN: eosinophil chemotactic proteins, *FGFBASIC: fibroblastic growth factor, basic, GCSF: granulocyte colony stimulating factor, GMCSF: ganulocyte-macrophage colony stimulating factor, *HGF: hepatocyte growth factor, IFNA: interferon alpha, IFNG: interferon gamma, IL01interleukin 1a, IL01b: interleukin 1b, IL01Ra: Interleukin 1 receptor antagonist, IL02: interleukin 2,* IL02R: interleukin 2 receptor, IL03: interleukin 3, IL04: interleukin 4, IL05: interleukin 5, IL06: interleukin 6, IL08: interleukin 8, IL09: interleukin 9, IL10: interleukin 10, IL12: interleukin 12, IL13: interleukin 13, IL15: interleukin 15, IL17A: interleukin 17A, IL17F: interleukin 17F, IL22: interleukin 22, CXCL10: C-X-C motif ligand 10, CCL2: C-C motif ligand 2, CXCL9: C-X-C motif ligand 9, CCL3: C-C motif ligand 3, CCL4: C-C motif ligand 4, *CCL5: C-C motif ligand 5, VEGF: vascular endothelial growth factor. *Difference in populations based on box plots.

The pre- and post-antivenom cytokines and chemokines were compared in **Figure 2**. There were changes in log values pre- and post-antivenom with increases in the median of the log of basic fibroblast growth factor (FGFBasic)(1.74 to 2.97), interleukin-2 receptor (IL02R)(3.04 to 4.50) and regulated on activation, C-C motif ligand chemokine (CCL5)(0.28 to 0.41) and a decrease in human growth factor (HGF)(6.24 to 5.85).

**Figure 3:**
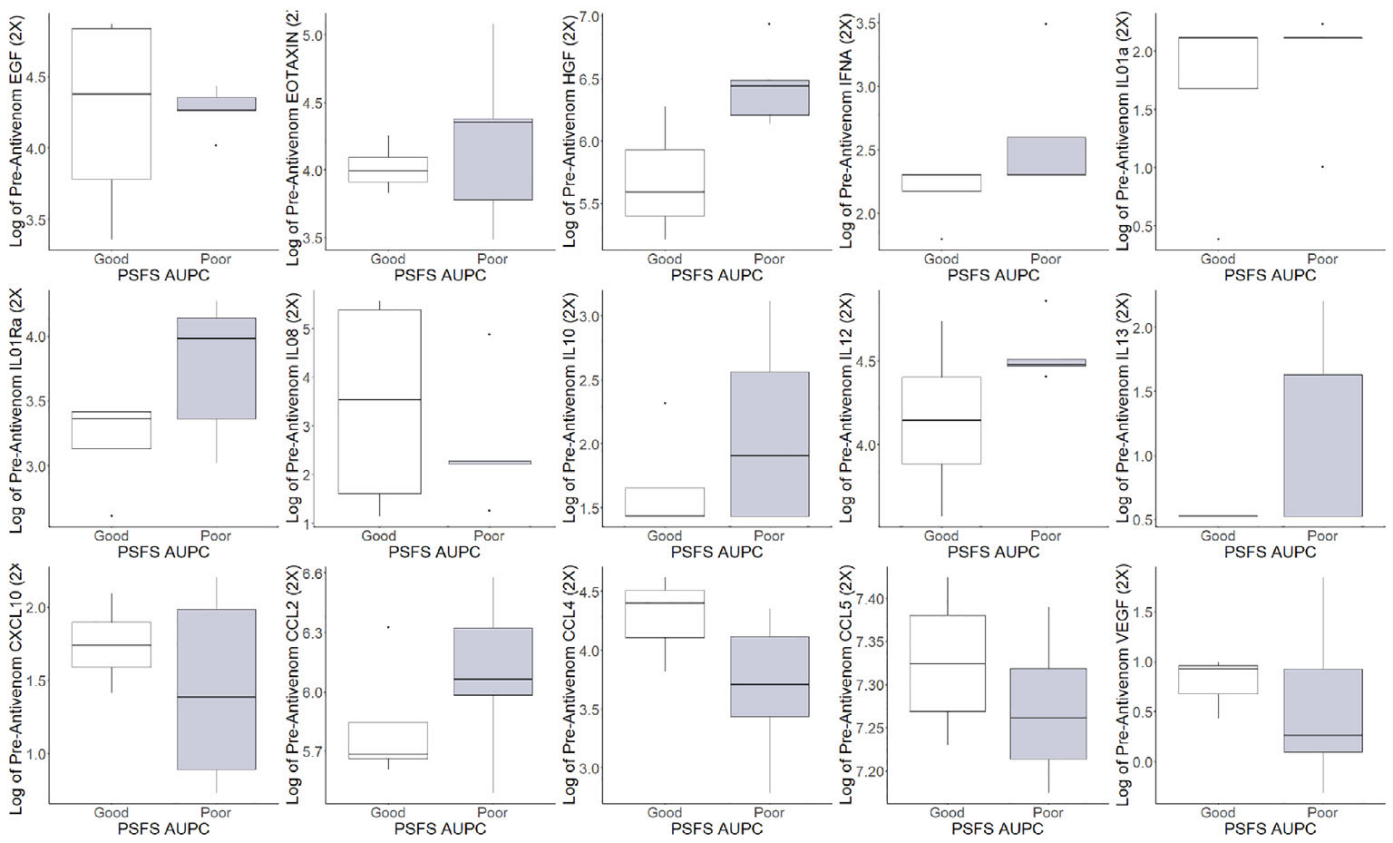
Pre-antivenom cytokines and chemokines values of patients with good and poor recovery. EGF: endothelial growth factor, *EOTAXIN: eosinophil chemotactic proteins, *HGF: hepatocyte growth factor, IFNA: interferon alpha, IL01a: interleukin 1a, IL01Ra: Interleukin 1 receptor antagonist, IL08: interleukin 8, *IL10: interleukin 10, *IL12: interleukin 12, IL13: interleukin 13, *CXCL10: C-X-C motif ligand 10, *CCL2: C-C motif ligand 2, *CCL4: C-C motif ligand 4, *CCL5: C-C motif ligand 5, *VEGF: vascular endothelial growth factor. *Difference in populations based on box plots.

**Figure 4:**
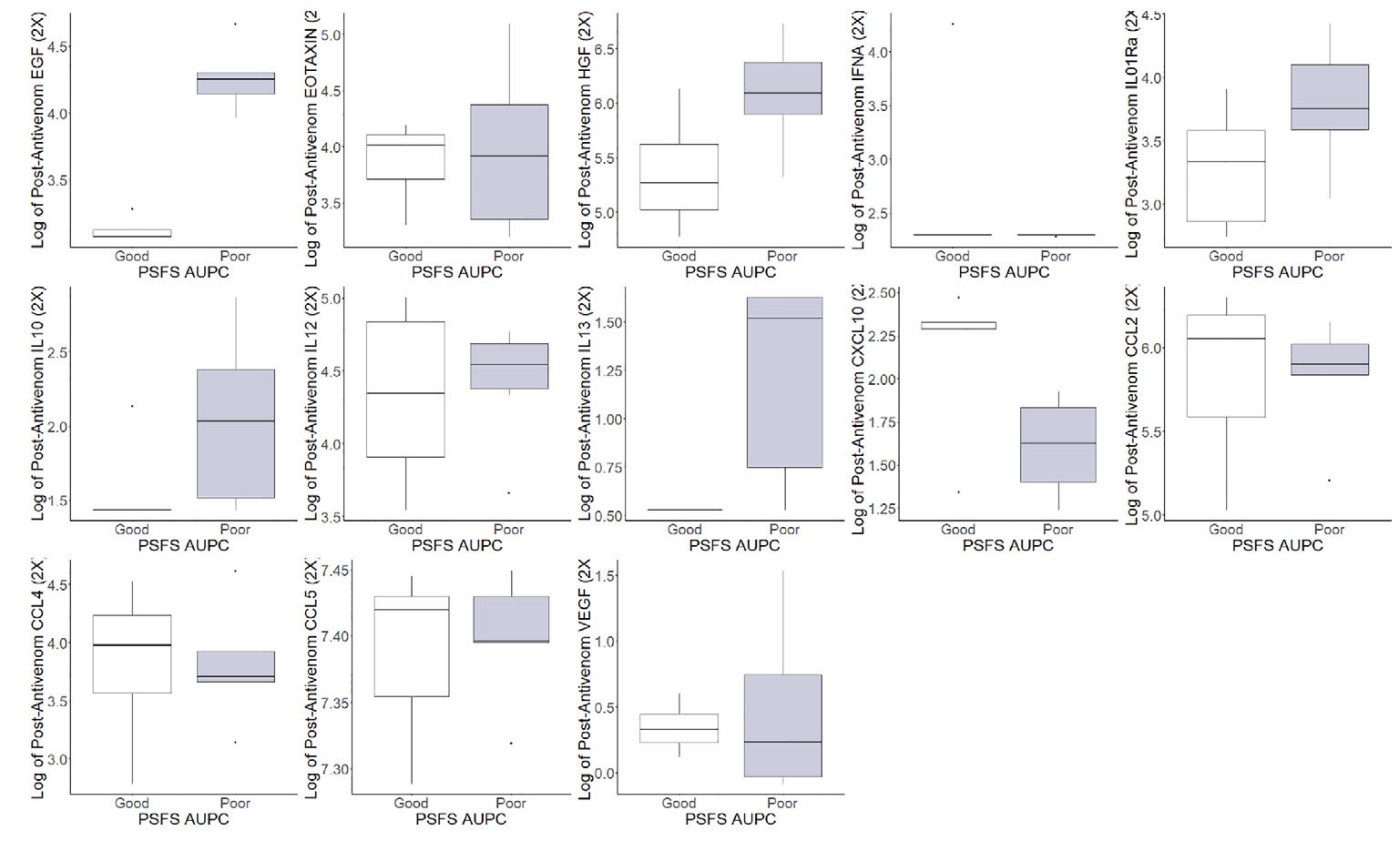
Post-antivenom cytokine and chemokine values of patients with good and poor recovery. *EGF: endothelial growth factor, EOTAXIN: eosinophil chemotactic proteins, *HGF: hepatocyte growth factor, IFNA: interferon alpha, *IL01RA: Interleukin 1 receptor antagonist, IL10: interleukin 10, *IL12: interleukin 12, IL13: interleukin 13, *CXCL10: C-X-C motif ligand 10, *CCL2: C-C motif ligand 2, *CCL4: C-C motif ligand 4, CCL5: C-C motif ligand 5, VEGF: vascular endothelial growth factor. *Difference in populations based on box plots.

The pre-antivenom individual cytokines and chemokines values were compared between good and poor functional recovery and are represented in **Figure 3**. In the pre-antivenom data, patients with poor recovery have higher log values of the eosinophil chemokine subfamily EOTAXIN (poor recovery median 4.35, good recovery median 3.99), HGF (6.44, 5.59), interleukin-1 receptor antagonist (IL01Ra) (3.98, 3.96), IL10 (1.90, 1.43), IL12 (4.47, 4.14), and C-C motif ligand 2 (CCL2) (6.06, 5.69). Patient with poor recovery had lower log values of CXCL10: C-X-C motif ligand 10 (1.39, 1.74), C-C motif ligand 4 (CCL4) (3.71, 4.40), CCL5 (7.26, 7.33), and vascular endothelial growth factor (VEGF) (0.26, 0.92). The post-antivenom individual cytokines and chemokines values were compared between good and poor functional recovery and are represented in **Figure 4**. Patients with poor recovery have higher values of endothelial growth factor (EGF) (poor recovery median 4.25, good recovery median 3.08), HGF (6.09, 5.27), IL01Ra (3.75, 3.33), and IL12 (4.54, 4.34) with lower values of CXCL10 (1.62, 2.29), CCL2 (5.90, 6.05), and CCL4 (3.71, 3.98).

### Prognostic Modeling Networks

**Figure 5:**
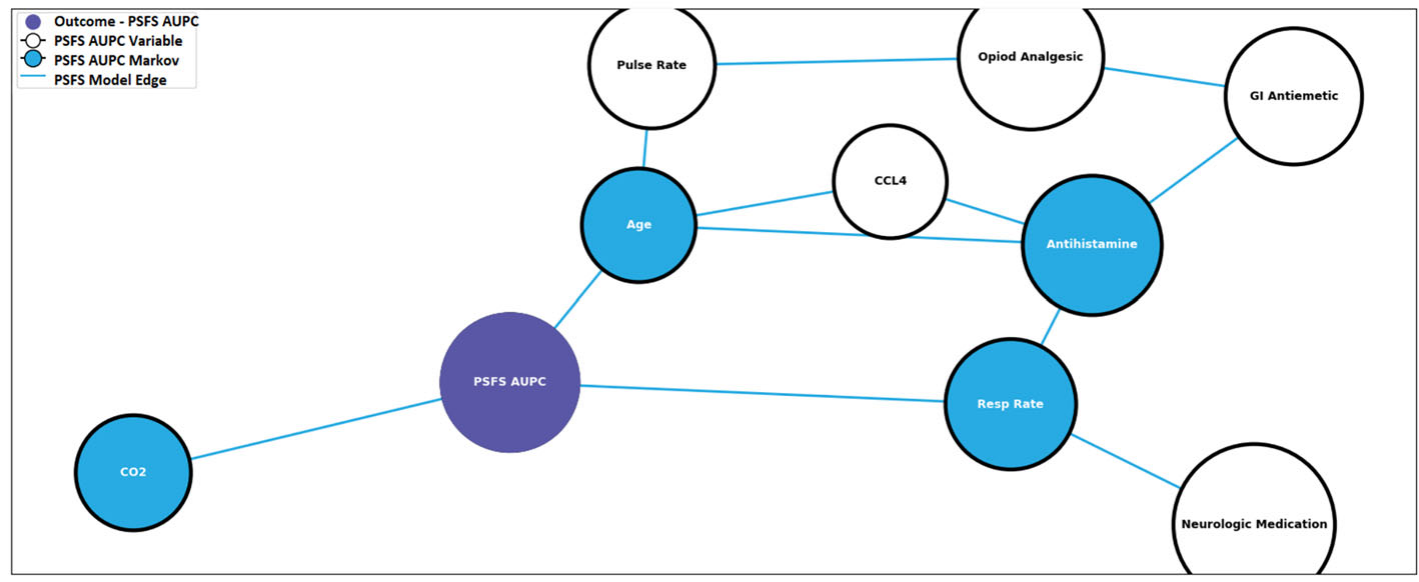
Pre-antivenom prognostic model predictive of recovery. PSFS AUPC: Patient-Specific Functional Scale Area Under the Patient Curve, CCL4: C-C motif ligand 4, GI: gastro-intestinal, CO_2_: carbon dioxide, Resp Rate: respiratory rate.

**Figure 6:**
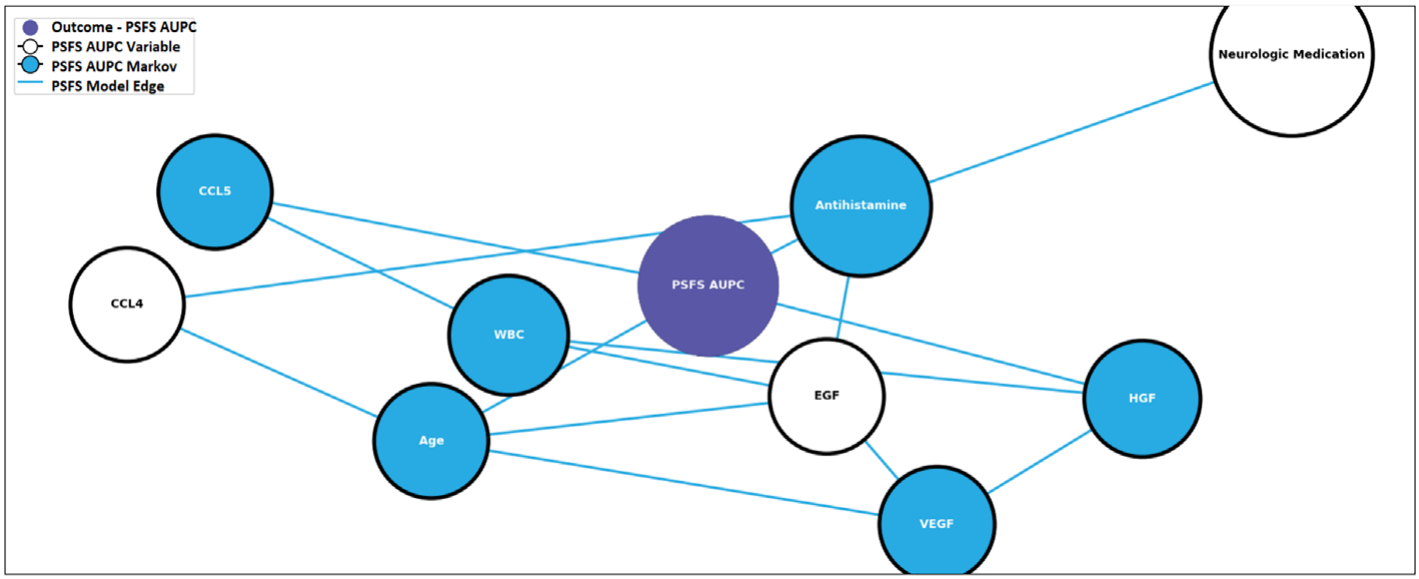
Post-antivenom prognostic model predictive of recovery. PSFS AUPC: Patient-Specific Functional Scale Area Under the Patient Curve, CCL4: C-C motif ligand 4, CCL5: C-C motif ligand 5, ECG: endothelial growth factor, HGF: hepatocyte growth factor, VEGF: vascular endothelial growth factor, WBC: white blood cell count.

In order to illustrate the effect antivenom has on the networks, the pre-and post-antivenom modeling networks are presented in **Figures 5** and **6**, respectively. In the pre-antivenom model, the variables most closely associated with the PSFS AUPC (i.e. recovery) are predominantly clinical features such as age, respiratory rate, CO2 (mostly bicarbonate) as measured by the basic metabolic panel, white blood cell count (WBC) and the need for antihistamines. These variables are also the most influential on the model, according to the Minimum Description Length (MDL) score. In the post-antivenom model, cytokines and chemokines are more fully incorporated into the model. The variables most closely associated with the PSFS AUPC are age, antihistamines, HGF, CCL5 and VEGF. The most influential variables are age, antihistamines and ECF. Within the limits of this small preliminary dataset, both the pre- and post-antivenom models perform well with AUCs of 0.87 and 0.90 respectively. Model stability was not assessed.

## Discussion

Using a preliminary data set from patients with SBE, we identified distinct pre- and post-antivenom patterns of clinical features combined with cytokines and chemokines that are associated with recovery. These findings are important as they demonstrate the feasibility of using this technique to develop a more predictive prognostic model than currently exists. Ultimately this could result in a clinical decision support tool capable of determining thresholds to treat with antivenom or other novel therapeutics and/or informing the need for escalating therapies. This individually tailored therapy approach holds the potential to most efficiently improve patient outcomes from SBE while preserving treatment for those likely to benefit.

The functional impact of SBE varies widely from patient to patient even within a given envenoming species. Specific SBE-related disabilities may impact different functions from work, to self-care, domestic life, social interactions, or civic life to name a few (62–64). By predicting and mitigating morbidity, we can maximize the economic, societal, and individual impact of SBE treatment. This is of particular importance in low-resourced areas of LMICs where the majority of SBE-associated negative consequences occur (5).

Our technique has already been used to enhance the care of other disease states such as infection and injury. In 2019, Gelbard et al. developed models using clinical data, cytokines, chemokines and growth factors that predicted severe sepsis and organ space infections following laparotomy for abdominal trauma (58). This modeling approach has expanded to combat trauma, where a predictive model composed of clinical features and immunologic biomarkers can accurately predict pneumonia in a predominantly blast-injured cohort of patients (59).

By expanding this technique to SBE, we have built on the prior work evaluating both the clinical features predicting severity and studies of the association of soluble biomarkers with severity (47, 48, 65, 66). In doing so, we are able to overcome some of the limitations of the initial forays into prognostication in SBE. For example, a multitude of basic clinical features have been assessed in predicting severe SBE (33, 34, 36, 37, 40, 43, 56, 67, 68). A number of these established clinical features, such as young age and time to care, are now known to increase the likelihood of a severe envenoming. However, none can adequately exclude severe SBE (40). Consequently, they have not been assessed as a tool to determine the need to treat or not to treat with antivenom. Additionally, the commonly applied laboratory testing have not even been demonstrated to confidently predict their own future trend (40, 56, 69). Finally, combinations of these clinical features and laboratory tests have been combined into a number of grading scales (42, 43). However, none of these have had comprehensive psychometric evaluations that assess their validity and reliability in predicting patient-centered outcomes.

Similarly, the associations of soluble immunologic biomarkers such as cytokines, chemokines and complement with acute findings of tissue injury are now being studied. In 2019, Ibiapina and colleagues evaluated the relationships between seventeen of these biomarkers and tissue injuries. Relationships were evaluated between biomarkers of healthy controls and patients with mild or severe tissue injury in *Bothrops asper* SBE. Important associations were established with individual biomarkers as well as classes of immune response polarizations such as Th-1,Th-2, and Th17. However, their results are limited by the lack of an established relationship between acute tissue findings they evaluated, such as swelling, blisters, or superficial necrosis, with important patient-centered outcomes such as functional recovery (48).

Our study also shows relationships with individual immunologic biomarkers and clinical outcomes. For example, Th-2 polarization-associated cytokines such as IL-10 and IL-13 were increased in patients with poor outcomes, as were the chemokines CXCL 10, and the growth factors EGF and HGF. Our preliminary data is not yet robust enough to reliably characterize the comprehensive nature of the immune response in SBE. However, for prognostication purposes, it appears that we can overcome prior limitations by using advanced machine learning models using clinical and immunologic laboratory features to accurately predict a well-validated, patient-centered, functional outcome.

In addition to prognostic model development, our data give us further insight into potential immunologic therapeutic targets in the sterile inflammatory disease state of SBE. For example, nonsteroidal anti-inflammatory drugs have been typically avoided in SBE due to their antiplatelet effects and the potential hemotoxicity of snake venom. However, the study of their beneficial impact on cytokine response and wound healing in trauma demonstrates the need to further study NSAIDs and immune-mediated outcomes in an appropriate SBE population (61, 70, 71). Ultimately, a comprehensive evaluation of individual biomarkers including their change over the natural history of SBE, their relationship with outcome, the impact of antivenom on their levels, their role in prognostic modeling, and the presence of existing therapeutics targeting the specific biomarker should all impact their candidacy as a viable therapeutic target.

Moreover, consistency of results will play an important role in this work. Interestingly, this study did not find a significant elevation of tumor necrosis factor alpha (TNF-alpha). Previous work has shown a relationship between increasing TNF and SBE (72–74). Our unanticipated finding may be due to the predominant species enrolled in this study, *Agkistrodon contortrix*, is known to typically have a less severe clinical course. However, this finding should be verified using other cytokine assays to better characterize its role compared to SBE from other species.

## Limitations/Future Directions

Our preliminary findings are limited by the small sample size currently available for analysis. This resulted in some atypical demographic patterns such a female predominant study population; whereas, a similar SBE population in our geographic region is typically slightly male predominant (75). This also limits our ability to perform more nuanced analysis such as evaluating outcomes based on the total amount of antivenom administered. Despite this, the models do perform well and the anticipated additional data can be used to refine the models and assess model stability. In order to establish prognostic models that would be usable in clinical practice the immunologic evaluation relies on cytokine and chemokine data that can be easily obtainable in a timely fashion. A more comprehensive immunologic evaluation such as complement levels, flow cytometry, transcriptomics, and venom associated molecular patterns (VAMPS) would give a more global picture of the immunologic response to SBE than is available in this preliminary analysis. Likewise additional clinical data such as presenting severity grade or time from bite to presentation could be incorporated into the model.

This study is limited to only a few species of Crotalinae snakes and the predominant species is known to have a less severe clinical course than others. Yet, our data demonstrate a wide distribution of recovery as measured by PSFS AUPC over 28 days. The heterogeneity of the disease itself and the utility of our chosen outcome measure allows us to discriminate between patient’s recovery and develop robust prognostic models. This technique can be easily applied to other Crotalinae snake species. Lastly, as this is preliminary work, we have not used the models to determine thresholds to treat nor assessed clinical efficacy of implementing this application.

## Conclusion

Pre- and post-antivenom networks of cytokines/chemokines and clinical features were associated with functional recovery measured by the PSFS AUPC over 28 days. With additional patient data, we can identify prognostic models using immunologic and clinical variables to predict recovery from snake envenoming.

## Data Availability Statement

The raw data supporting the conclusions of this article will be made available by the authors, without undue reservation.

## Ethics Statement

The studies involving human participants were reviewed and approved by Duke University School of Medicine Institutional Review Board. The patients/participants provided their written informed consent to participate in this study.

## Author Contributions

All authors listed have made a substantial, direct, and intellectual contribution to the work and approved it for publication.

## Funding

Grant funding for this research: United States Department of Defense. USUHS HT9404-13-1-0032 and USUHS HU001-15-2-0001.

## Conflict of Interest

ES was employed by DecisionQ. CG receives grant funding from BTG Specialty Pharmaceuticals.

The remaining authors declare that the research was conducted in the absence of any commercial or financial relationships that could be construed as a potential conflict of interest.
